# Filiform polyposis presenting with rapid growth and severe anemia case report

**DOI:** 10.1016/j.ijscr.2022.107771

**Published:** 2022-11-01

**Authors:** Abdullah alkhuzaie, Waed jameel, Noran sultan, Mohammed aldosari

**Affiliations:** a Department of General Surgery, King Abdulaziz General Hospital, Makkah, Saudi Arabia; b Department of Histology, King Abdulaziz General Hospital, Makkah, Saudi Arabia

**Keywords:** Giant inflammatory polyposis (GIP), Inflammatory bowel disease (IBD), Ulcerative colitis (UC), Colorectal cancer (CRC), Filiform polyposis (FP), Case report

## Abstract

**Introduction:**

Giant inflammatory polyposis (GIP) is a rare manifestation of inflammatory bowel disease (IBD), and it is described as a worm-like projection of 1.5 cm or more in diameter with unclear pathogenesis. GIP may be related to the severity of IBD. GIP presents with a wide range of symptoms, including crampy abdominal pain, anemia, and intestinal obstruction. The histopathology of GIP is a polyp lined by normal colonic mucosa with superficial ulceration that may show mild crypt distortion.

**Case report:**

Our case reports a patient with ulcerative colitis diagnosed via colonoscopy and histopathology 10 months before presenting with severe anemia due to lower gastrointestinal bleeding. Colonoscopy showed GIP obstructing the descending and sigmoid colon, and total colectomy showed the entire colon full of worm-like polyps up to 14 cm the longest polyp.

**Discussion:**

Giant inflammatory polyposis which is seen in 17 % of UC with active colitis, as seen in our patient, the histopathology component was acute in addition to chronic, which formed within 6 months. According to the data in the literature, the average duration reported for formation since diagnosis with UC is approximately 3 to 276 months. With a length up to 16 cm, the sigmoid colon is the most common site.

**Conclusion:**

Surgical intervention is indicated for filiform polyposis (FP) if it is complicated, such as bleeding or obstruction, which is reported in a few cases. However, there are no clear guidelines for surgical intervention for complicated FP, but the safest method is to tailor the management according to the guidelines for each underlying disease.

## Highlights

• Patient presented with complicated filiform polyposis and aggressively rapid caused severe anemia required blood transfusion.
• It’s a rare manifestation of IBD, which require closer follow up of the patients and consider measures before complications occurs.
• The case report is rare within the literature this could add value to build up our knowledge and approach to similar cases.

## 1 Introduction

This case report has been reported in line with the SCARE 2020 criteria [1]. Giant inflammatory polyposis (GIP), also named giant pseudopolyposis and filiform polyposis, is an uncommon pathological feature of inflammatory bowel disease (IBD) [2]. GIP is identified as multiple worm-like projections measuring 1.5 cm or more in length or diameter in the colon [3]. GIP can reach up to 100 in number [11]. GIP may occur in Crohn's disease (CD), ulcerative colitis (UC) or indeterminate colitis (IC) [2]. Most of the review studies reported that GIP was higher in UC than CD [3], [4].

GIP is more common in males than females, and the age group is 8 to 68 years old [4].

Clinical manifestations of GIP are nonspecific and may present as gastrointestinal bleeding, cramping abdominal pain, intestinal obstruction, or asymptomatic as well as incidental symptoms during colonoscopy [5].

We present a case of UC with a rapid growing GIP within 10 months that caused severe anemia.

## 2 Case report

A 58-year-old Saudi male known case of diabetes, patient presented to the emergency department at King Abdulaziz General Hospital, Makkah, Saudi Arabia, complaining of fatigability and dizziness for the past two days associated with two weeks cramping abdominal pain and multiple episodes of bloody diarrhea. No history of surgical procedures or drug abuse.

The patient was diagnosed with UC 10 months ago and he was non complaint to his treatment. A positive history of an intermittent abdominal pain and cramping within the last 2-years associated with bloody diarrhea that was significant as patient needed to receive blood transfusions. The final diagnosis was made after colonoscopy, which showed chronic relapsing ulcerative colitis.

Examination showed a soft abdomen, nontender, nondistended, with no guarding or rebound tenderness. Digital rectal examination revealed stool mixed with blood. His laboratory investigation was unremarkable except for the hemoglobin (hgb) 3.1 g/dL, and leukocytosis 8.3 10^9/L.

The patient was admitted, and blood transfusion was initiated. After stabilization of the patient hgb, a search for the cause was started.

The upper endoscopy was unremarkable on the other hand the Colonoscopy described hyperemic rectal and sigmoid colon mucosa with ulcerated surface and multiple worm-like polyps started 20 cm from the anal verge, the scoop reached 38 cm from the anal verge to the descending colon and could not progress farther due to an obstructed colon with worm-like polyps (Pic 1). Multiple biopsies were taken.

**Pic 1:**
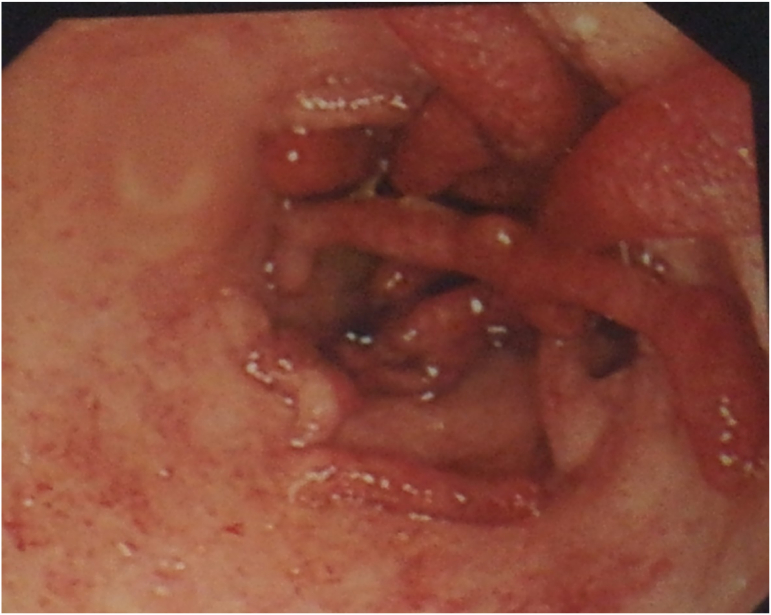
Colonoscopy image.

The histopathologist verified the diagnosis, a chronic active colitis consistent with ulcerative colitis. An abdominal computed tomography scan (CT) was performed with Intravenous and oral contrast revealed the loss of the ascending and sigmoid colon with wall thinking and multiple tubular dissecting intraluminal filling defects in the ascending and sigmoid colon, likely polyps with filiform patterns (Pic 2).

**Pic 2:**
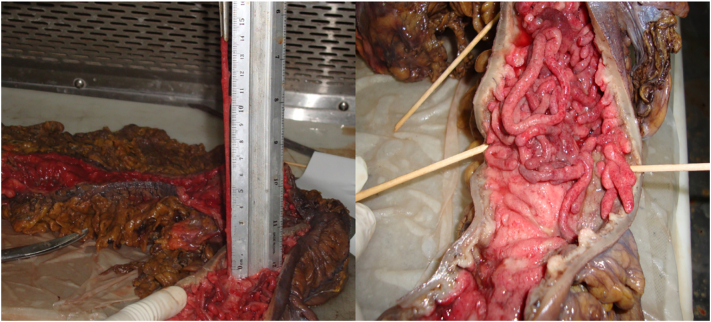
Surgical specimen.

A total colectomy with ileo-rectal anastomosis with circular end-to-end stapling devices as performed with no intra operative complications by the main surgeon Dr. Alkhuzaie.

Postoperatively the patient had no complications. He started an oral diet gradually from day one tolerated with no complains furthermore he was discharged three days later, the patient seen in the clinic 2 weeks later and one year later without any complications.

Regarding the colon specimen, grossly a display of multiple projections with a worm-like shape, and the longest one was 14 cm (Pic 2). The final histopathology report showed neutrophil infiltration of the mucosa and submucosa with crypt abscesses, and no signs of dysplasia.

## 3 Discussion

There are several hypotheses for the formation of inflammatory polyps, including severe mucosal inflammation that occurs due to the IBD, which overgrows the mucosa, after the inflammation subsides, due to fecal stream traction or repeated inflammation and healing of mucosa during the healing period [4], [6], [7], this is seen in 17 % of UC with active colitis [8]. As in our patient, the histopathology component was acute on top of chronic UC, which formed within six months only. According to the data in the literature, the average duration reported for formation since diagnosis with UC is approximately 3 to 276 months [5]. With a length up to 16 cm [9], the sigmoid colon is the most common site [7], [10], which is consistent with the fact that 30–50 % of UCs are confined to the rectum and sigmoid colon [11]. The histopathological appearance of FP is generally normal to acute or chronic inflammatory colonic mucosa [10], [12].

Moreover, the risk of colorectal cancer (CRC) is higher in UC patients compared to the general population, and it is increased with extensive colitis, primary sclerosing cholangitis, family history of colorectal cancer, chronic colonic mucosal inflammation and post inflammatory polyps [11], [13]. FP is considered an independent risk factor for malignancy [4].

The prevalence of CRC in UC decreased over the past few decades according to a Danish study, and it is 1.7 % compared to the general population [14]. This reduction referred to the increase in the colonoscopy surveillance in UC patients [15]. Despite the risk of malignancy in UC, the question is whether FP is premalignant or not, and is it considered a risk factor for malignancy? [10] As in our case, the histopathology did not show any neoplasia or dysplasia, as documented in many case reports, because most FP is found incidentally and does not need further management, except usual surveillance [12]. Notably, only four case reports in the literature linked FP with malignancy, two in IBD patients and the other two in non-IBD patients [7], [10], [16], [17].

Surgical intervention is indicated for FP, if it is complicated, such as bleeding or obstruction, which is reported in a few cases [2], [4], [5], [6]. However, there are no clear guidelines for surgical intervention for complicated FP, but the safest methodology is to follow the guidelines per case and the underlying disease, as seen in this case, an emergency surgical option was indicated with a total abdominal colectomy with end ileostomy [11].

## 4 Conclusion

Inflammatory polyps have no specific management except in complicated cases such as in bleeding or obstruction, which have been reported in a few cases [2], [4], [5], [6]. Otherwise, colonoscopy surveillance with multiple biopsies may be considered a safe approach [12] because the risk of malignancy transformation is not clearly understood, which requires further studies and research.

## Ethical approval

All authors have NIH certification and as case report need only patient consent.

## Funding

No funding needed in our case report writing.

## CRediT authorship contribution statement

Dr. Waed Saeed Jameel: Main author, Literature review and writing the main manuscript.

Dr. Noran Abdurazzaq Sultan: Co-author, Wrote the case report part and assist in writing the manuscript.

Dr. Abdullah Mosleh Alkhuzaie: Co-author, The consultant in charge of the case during admission, also reviews and adjusts the manuscript.

Dr. Mohammed Saeed Aldosari: Co-author, review the manuscript and the pathology sample.

## Guarantors

Dr. Waed Saeed Jameel: Main author, Literature review and writing the main manuscript.

Dr. Noran Abdurazzaq Sultan: Co-author, Wrote the case report part and assist in writing the manuscript.

## Registration of research studies

Not applicable.

## Declaration of competing interest

The authors confirm that there are no known conflicts of interest.
